# Acetaminophen for self-reported sleep problems in an elderly population (ASLEEP): study protocol of a randomized placebo-controlled double-blind trial

**DOI:** 10.1186/1745-6215-15-10

**Published:** 2014-01-07

**Authors:** Esther MM van de Glind, Lotty Hooft, Linda R Tulner, Joke HM Tulen, Ingeborg MJA Kuper, Hans L Hamburger, Sophia E de Rooij, Barbara C van Munster

**Affiliations:** 1 Department of Internal Medicine, Geriatrics Section, Academic Medical Center, P.O. Box 22660, Amsterdam, 1100 DD, The Netherlands; 2 Dutch Cochrane Centre, Academic Medical Center, University of Amsterdam, Amsterdam, The Netherlands; 3 Department of Geriatric Medicine, Slotervaart Hospital, Amsterdam, The Netherlands; 4 Department of Psychiatry, Erasmus MC, Rotterdam, The Netherlands; 5 Department of Clinical Neurophysiology and Amsterdam Center for Sleep-Wake Disorders, Slotervaart Hospital, Amsterdam, the Netherlands; 6 Department of Geriatric Medicine, Gelre Hospitals, Apeldoorn, The Netherlands

**Keywords:** Acetaminophen, Geriatrics, Protocol, RCT, Sleep

## Abstract

**Background:**

The prevalence of sleep disorders increases with age. Sleep disorders may have serious health implications and may be related to serious underlying diseases. Many older people use hypnotics, like benzodiazepines, although these medications have serious side effects and often lead to habituation. Acetaminophen is one of the most frequently used off-label drugs for sleep disorders, although little is known about its effects. Our objective is to investigate whether acetaminophen is effective in treating self-reported sleep disorders in older people.

**Methods/Design:**

Participants, aged 65 years or older (*n* = 150), who have sleep disorders will be randomized for treatment with either acetaminophen 1000 mg or placebo, once daily at bedtime in a double-blind design. Eligible patients should be able to give informed consent, should not be cognitively impaired (Minimal Mental State Examination (MMSE) score ≥ 20), should not have pain, and should not use acetaminophen on a regular basis because of pain complaints. The study will take three weeks to complete. During these three weeks, the participants register their sleep behavior in a sleep diary. The participants will use the study medication during the second and third week. The primary endpoint will be the self-reported sleep disorders at the end of week three, as measured by means of the Insomnia Severity Index (ISI). To validate these subjective sleep parameters against objectively measured indices of the sleep-wake pattern, we will measure the periods of wakefulness and sleep in a subgroup of participants, using an actigraph worn on the wrist during the entire study period.

**Discussion:**

The proposed study will contribute to our knowledge about the treatment of sleep disorders in an older population. There is a need for treatments for sleep disorders without serious adverse effects. Acetaminophen might be a simple and inexpensive alternative for the regimes that are currently used with older people.

**Trial registration:**

The Netherlands National Trial Register NTR2747.

## Background

The prevalence of sleep disorders is high in older subjects. In the Established Populations for Epidemiologic Studies of the Elderly (EPESE) study, involving 9,282 community-dwelling persons aged 65 and older, 50% reported sleep complaints, of which 25% had insomnia [1]. In a study of older patients in 11 primary care practices (*n* = 1503), the most commonly reported sleep-related complaints were difficulty sleeping (45%), snoring (33.3%), and excessive daytime sleepiness (27.1%) [2]. Subjects with insomnia report a negative impact on quality of life [3].

There are various hypotheses as to why older persons suffer from sleep disorders. Single or multiple medical problems, such as depression, cardiovascular diseases, and pulmonary problems can interfere with a good night’s rest. In addition, older people often use medication that causes insomnia, for example beta blockers and psychopharmacological drugs. Only a minority of older people suffer from primary insomnia [4].

The best treatment for sleep disorders remains a topic of discussion. There is evidence for a positive effect of cognitive behavioral interventions for sleep disorders in older people [5,6], but these are time-consuming and dependent on patient motivation. Some authors state that bright light therapy might help, especially in the case of a dysregulation of the circadian rhythm, but as yet there have been no randomized trials [7]. There is a lack of evidence from well-designed trials of the effectiveness of physical exercise for the treatment of sleep problems in healthy older people [8]. The most common way of treating sleep disorders is the pharmacological approach [9]. Many older people use a sedative benzodiazepine as a hypnotic, although they are at risk of harmful side effects because of age-related pharmacodynamic and pharmacokinetic changes [10]. Adverse events, such as falling [11,12], cognitive impairment [13], and drug dependence [14], and even an increased risk of death [15] are reported. Considering the health impact, the complexity and the high prevalence, sleep disorders are an important area of investigation and simple treatments with fewer side effects are urgently needed.

In geriatric clinical practice, we have noticed that older patients use acetaminophen for sleep problems without having underlying pain complaints. In a survey of 176 older people, 48% stated that they used nonprescription products for sleeping problems. Of the individuals who had used a nonprescription product, 19% used acetaminophen [16]. Although we did not find any trials or observational studies in the literature that report the effect of acetaminophen on sleep disorders, there are some ideas as to why this medication might have a positive effect. People who sleep better on acetaminophen may have unrecognized pain complaints. Another possible hypothesis is that acetaminophen, after metabolization in the brain, reinforces the activity of the cannabinoid receptors, which in turn reinforce the activity of the serotonergic system [17,18]. Also, there may be a relationship with body temperature [19], or its purported effect could be mainly placebo.

This paper describes the design of the study named ASLEEP (Acetaminophen for SLEep Problems in Elderly Patients), a trial currently in progress. The aim of this trial is to investigate whether acetaminophen has an effect on self-reported sleep disorders in older people, as measured by a score on a validated sleep questionnaire, the Insomnia Severity Index (ISI) [20]. Also, to validate the subjective sleep parameters against objectively measured indices of the sleep-wake pattern, we aim to study the effects of acetaminophen on periods of wakefulness and sleep, as measured by actigraphy in a subgroup of our participants [21].

## Methods/Design

This study is a randomized, placebo-controlled, double-blind, national, multicenter study with a duration of three weeks. This study will be carried out in three teaching hospitals in the Netherlands: the Academic Medical Center, the Gelre Hospitals, and the Slotervaart Hospital. The study population will consist of people aged 65 years and older suffering from sleep disorders, defined as one or more of the following symptoms: difficulty falling asleep, difficulty maintaining sleep or early awakenings without being able to fall asleep again, with a frequency of at least three nights a week, during at least three consecutive weeks [22]. Participants may be patients that visit one of the participating hospitals’ outpatient clinics, or they may be subjects who are recruited after advertising in local newspapers. The visitors of the outpatient clinic will be enrolled during their visit; the people who respond to the advertisements will be invited to come to one of the participating hospitals for enrollment.

Eligible patients should have a score of five points or more on the Pittsburgh Sleep Quality Index (PSQI) [23]. This is a validated instrument that assesses sleep quality and disturbances over a 1-month time interval. A global PSQI score greater than 5 yielded a diagnostic sensitivity of 89.6% and specificity of 86.5% in distinguishing good and poor sleepers [23]. Further inclusion criteria are: a score of 20 points or more on the Minimal Mental State Examination (MMSE) [24] and participants must be willing and medically able to receive therapy according to the protocol for the duration of the study. In addition, participants must be able to give informed consent themselves.

Exclusion criteria are the inability to speak, understand or write Dutch; the inability to follow the study procedures, as assessed by the researcher; alcohol intake of more than four units daily; the concomitant use of acetaminophen (>1000 mg daily); pain complaints resulting in a pain score of 6 or higher on a visual analog scale; impaired liver function; suicidal tendencies; and participation in other sleep trials. In addition, patients with a life expectancy of less than three months, according to the attending physician, or with a sleep problem due to a medical or somatic reason (a suspicion of obstructive sleep apnea syndrome, restless legs, delirium, or a depression necessitating antidepressants) are excluded.

Demographic data, medical history, and medication use are recorded at baseline. The cognitive status is assessed with the MMSE [24] and functional status is assessed with the Katz-15 Activities of Daily Living index [25]. The severity and number of comorbidities is scored with the Charlson comorbidity index [26]. Possible confounding factors, like the use of comedication, other sleeping pills, and the use of alcohol and coffee are registered for all patients.

During the first week, participants only register their sleep patterns. In the second and third weeks, they also take either 1000 mg acetaminophen in two tablets of 500 mg each, or two tablets of an exact matching placebo, once per day at bedtime. Study medication is packaged in small bottles labeled according to Good Manufacturing Procedure guidelines. The medication is provided in sequentially numbered containers according to the randomization list. In the sleep diary, patients register if they had taken the study medication the night before.

Participants are allowed to continue their own prescriptions, including their sleeping pills. They are asked not to use newly prescribed sleeping pills. However, sometimes it may happen that participants feel they cannot sleep without using a sleeping pill in addition to the study medication. If this happens, they are asked to register this in the sleep dairy. Should it happen that patients unexpectedly start a sleeping pill, they will no longer receive the study medication, but data collection will be continued.

Participants who need acetaminophen as a painkiller during the study period will be prescribed this as usual and will no longer receive the study medication. However, use of acetaminophen and not being able to stop this medication during the study period is a reason for exclusion. Also, patients who use acetaminophen as a sleeping pill are asked to stop the medication for the 3-week study period, that is, both for the baseline data collection and for the period that they use the study medication. In the case of an unexpected need for acetaminophen, despite the precautions taken in the enrollment procedure, participants should stop using the study medication, because of a risk of overdose. However, the data collection will be continued in these cases and we will perform both an intention-to-treat and a per-protocol analysis.

The primary outcome measure will be the difference in score on the ISI [6,27], as completed at the end of the first and third weeks. Secondary outcomes will be the use of extra sleeping pills and objective sleep parameters, registered by means of an Actiwatch [21,28]. The Actiwatch is a wristwatch-like device, worn on the nondominant hand, which records the motion for each epoch (typically 30 to 60 seconds) for several weeks. A subgroup of 25 patients enrolled in the Slotervaart Hospital will wear the Actiwatch for 24 hours a day during the three-week study period. In our trial, the Actiwatch Spectrum device (Philips Respironics) will be used. Movements are recorded in 1-minute epochs. The raw activity scores will be translated to sleep-wake scores for each epoch, based on standard algorithms, which are part of the proprietary software that will be used to analyze the data. In addition to this, all participants will record in a sleep diary the time they get into and out of bed, the estimated time they fall asleep and wake up in the morning and the number of awakenings during the night. Furthermore, patients will register a daily pain score by means of a 10 cm visual analog scale. In addition, they will rate their sleep quality with a score of 0 to 10. The combination of the actigraphy data and the sleep diary will be used to estimate the following sleeping and waking endpoints: total sleep time (minutes sleep between bedtime and waking time), sleep efficiency (percentage of time asleep while in bed), sleep onset latency (minutes between bedtime and the first block of consistent sleep scored epochs after bedtime), time awake after sleep onset (minutes awake between sleep onset and waking time), number of long wakeful episodes and number of daytime nap episodes [21]. The Actiwatch Spectrum has an ‘off wrist detection’ feature that will be used to exclude daytime data if the participant takes the actigraph off for more than 10% of the wake period [29].

The risks associated with this study consist of the possible adverse effects of acetaminophen. This is widely used as an analgesic and has proved to be effective and safe; therefore, the occurrence of side effects is not likely. However, all adverse effects will be registered according to Good Clinical Practice guidelines. During the study, patients will be contacted by phone to evaluate possible side effects.

An independent statistician provided a computer-generalized randomization list, using block randomization of ten participants per block. This list was stratified by study center. The trial-pharmacist is the only one in possession of the randomization list. The researcher who enrolled the patients is not aware of the assignment of the participants. Subject enrollment started on 1 July 2011.

This study will be carried out in compliance with the Helsinki Declaration. The Medical Ethics Committee of the Academic Medical Center has approved the study design, protocol, and informed consent procedures. The executive boards of the other participating centers, the Slotervaart Hospital and Gelre Hospitals, have provided local feasibility approval.

### Power analysis

The primary endpoint will be a reduction in subjective sleep disorders, as reflected in a lower score on the ISI at the end of the third week compared with the score after the first week. From the literature, a prevalence of sleep disorders of 30% to 50% is reported. Group sample sizes of 75 per group achieve 80% power to detect a difference of three points on the ISI between the null hypothesis that both group mean differences are zero and the alternative hypothesis that the mean difference of the intervention group is three, with an assumed group standard deviations of 6.5. This calculation is based on data from a study in which a group of patients with insomnia were treated with either eszopiclone or placebo [30].

### Statistics

Data will be primarily analyzed according to the intention-to-treat principle. The outcome measures will be tested by using *t* tests and Mann–Whitney tests for continuous variables and by using chi-squared tests for categorical outcomes. The data from the actigraphy and the sleep diary will be analyzed as continuous variables, either with an independent *t* test or with a Wilcoxon rank-sum test. We will perform a subgroup analysis for patients using sleeping pills at baseline and those who do not. If needed, we will adjust for the use of add-on sleeping pills. Analyses will be performed with the program Statistical Package for the Social Sciences (SPSS) version 18.

## Discussion

Sleep disorders are a major problem in older people, and are associated with comorbidities and high costs. To achieve good external validity, we intend to include patients with all comorbidities and applied minimal exclusion criteria. Older patients are often excluded from clinical trials [31,32]. An additional benefit of this research, which focuses on older patients, may be an insight into the challenges and methods for performing research with older patients. The proposed study will contribute to our knowledge about the treatment of sleep disorders in an older population by providing an alternative for the traditional hypnotics. There is a need for treatments for sleep disorders without serious adverse effects. Acetaminophen could potentially fill this gap, as it may be a promising simple and cheap alternative for regimes that are currently used with older people.

## Trial status

The ASLEEP trial is currently in progress; patient recruitment started on 1 July 2011 and was finished in July 2013.

## Abbreviations

ASLEEP: Acetaminophen for SLEep Problems in Elderly Patients; EPESE: Established populations for epidemiologic studies of the elderly; ISI: Insomnia severity index; MMSE: Minimal mental state examination; PSQI: Pittsburgh sleep quality index.

## Competing interests

The authors declare that they have no competing interests.

## Authors’ contributions

EMMvdG is responsible for the project organization and drafted the initial manuscript. BCvM and LH conceived and designed the study, supervised manuscript preparation, and obtained funding. SEdR is the principal investigator for this project. JHMT and HLH will be involved in the analysis of the actigraphy data. SEdR, LRT, IMJAK and HLH, Apologies, I think this was a typo contributed to data collection. All authors critically reviewed the draft and approved the final manuscript.
